# Biomimetic Adhesion/Detachment Using Layered Polymers with Light‐Induced Rapid Shape Changes

**DOI:** 10.1002/anie.202503748

**Published:** 2025-04-04

**Authors:** Youfeng Yue, Yasuo Norikane, Eiji Nishibori

**Affiliations:** ^1^ Core Electronics Technology Research Institute National Institute of Advanced Industrial Science and Technology (AIST) Tsukuba 305–8565 Japan; ^2^ Faculty of Pure and Applied Sciences and Tsukuba Research Center for Energy Materials Science (TREMS) University of Tsukuba Tsukuba 305–8571 Japan

**Keywords:** Crosslinked polymer networks, Nanoscale layered structures, Reusable films, Smart adhesion, Smectic liquid crystals

## Abstract

Geckos achieve rapid and efficient adhesion and detachment on various surfaces within milliseconds due to the hierarchically structured fibrillar architecture of their toe pads. Extensive research has focused on developing adhesive materials that mimic these micro‐nano structures. However, no conventional adhesives have matched the performance of their natural counterparts, which are both non‐degrading and self‐cleaning. Here, we develop a chemically crosslinked polymer film with a nanoscale layered structure that exhibits high‐speed switching of mechanical motions (expansion and contraction) in equation both forward and reverse directions through controlled ultraviolet (UV) irradiation. Under UV light on/off switching, the film shows reversable shape changes in the direction perpendicular to the molecular alignment. These photoresponsive molecular movements in the film is demonstrated for remote control in a smart adhesion system with rapid responsiveness and high reproducibility. The films respond to UV light to release picked‐up objects and immediately regain their adhesion when the UV light ceases. Additionally, we propose the working principles and mechanisms of these reusable adhesive films, providing new insights into the development of smart soft materials. The material's rapid deformation, high responsiveness, and flexibility make it a promising candidate for light‐controlled object transport and remote‐controlled robotics.

## Graphical abstract

Geckos achieve rapid adhesion and detachment through their hierarchically structured toe pads. Inspired by this, we developed a crosslinked polymer film with a nanoscale layered structure that undergoes high‐speed, reversible expansion, and contraction under UV light. This allows remote‐controlled adhesion switching, enabling rapid object pick‐up and release. This work offers insights into smart adhesives for soft robotics and object transport.

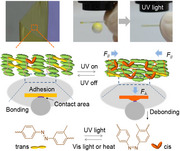

## Introduction

Insects, such as ants, possess adhesive toe pads composed of numerous branching protofibers oriented perpendicularly to the surface. These fine structures facilitate intimate contact with various substrates, enabling strong adhesion across diverse surfaces (such as hydrophobic, hydrophilic, rough, smooth, dry, or wet surfaces). Notably, their sticky pads can rapidly expand the contact area by pulling their legs centripetally, enabling their response to sudden mechanical perturbations, such as strong winds and raindrops. The short timescale of this response precludes any neuromuscular control and is more likely caused by the mechanical responses of the special cuticle of the toe pad.^[^ ^1^ ^]^ Changes in the orientation of the protofibers correlated with a change in the contact area. The pulling of the legs reduces the angle of the protofibrils and thus helps the lateral and longitudinal expansion of the contact area.^[^ ^1^ ^]^

Similarly, when facing threats, insects reduce their contact area to quickly detach from surfaces and escape. This dynamic control of the adhesive contact area has been observed in ants, which can adjust their contact areas both actively and passively. These mechanical “prereflexes” are not limited to ants as preliminary findings suggest similar reflex responses in geckos.^[^ ^2^, ^3^ ^]^ For example, geckos adjust the angle of their setae by changing the shape of the adhesive pads, achieving immediate detachment when the angle between the pads and the substrate is beyond 30°.^[^ ^3^ ^]^ These phenomena have inspired scientists across various fields to develop bioinspired adhesive materials. Designing a material that dynamically triggers mechanical motion and modulates the contact area in response to external stimuli could facilitate rapid adhesion/detachment switching. However, light‐induced, abrupt shape changes enabling fast adhesion/detachment within a single polymer network have been rarely demonstrated in the literature.

The use of polymers in bioinspired microstructured adhesives remains challenging. For instance, previous attempts to control adhesion using liquid–crystalline elastomers (LCEs) have relied on the thermoresponsive (or light‐induced thermal) properties of LCEs.^[^ ^4^, ^5^, ^6^ ^]^ These approaches required stimuli to reach high temperatures (50 °C–90 °C) to induce the phase transition of liquid crystals, thereby restricting the applicability of the material in daily life. Several successful examples of using small molecules or azopolymers as adhesives through light‐triggered solid‐to‐liquid phase transitions have been reported.^[^ ^7^, ^8^, ^9^, ^10^, ^11^, ^12^, ^13^, ^14^ ^]^ However, achieving fast and clean detachment in these light‐responsive systems remains difficult. Additionally, some adhesive materials require complex pillar‐ or mushroom‐like microstructures, increasing both the cost and complexity of adhesive manufacturing.^[^ ^15^, ^16^, ^17^, ^18^ ^]^ Some pioneering studies emphasis that an efficient switching of attachment and detachment requires gecko‐like orienting motions of the seta and the manipulation of the normal and lateral surface forces by the toes, rather than just simply using pillar‐like structures without considering the mechanisms of attachment and detachment.^[^ ^2^ ^]^

In this work, we design and synthesize a photocontrollable polymer film with dynamic adhesive and detachment capabilities. The film consists of poly (dodecyl glyceryl itaconate) (PDGI) as aligned molecules, chemically crosslinked by long‐alkyl‐chain photoisomerizable azobenzenes. Upon UV irradiation, the polymer network generates localized internal forces, causing expansion and reducing contact area with adherends, enabling reversible on‐demand bonding and debonding. Notably, this film achieves rapid detachment and quickly restores its initial adhesion state once UV irradiation ceases, allowing for reusable adhesives controlled by a single stimulus. Furthermore, a light‐controlled pickup–delivery–release system for transporting 3D solid objects has been developed. The working principle and mechanism have been proposed. This work offers insights into soft robotics and conceptual light‐controllable adhesion systems, with potential applications in pick‐and‐drop systems, reusable adhesives for daily use, and advanced technologies, such as microelectronics and space applications.

## Results and Discussion

**Figure 1:**
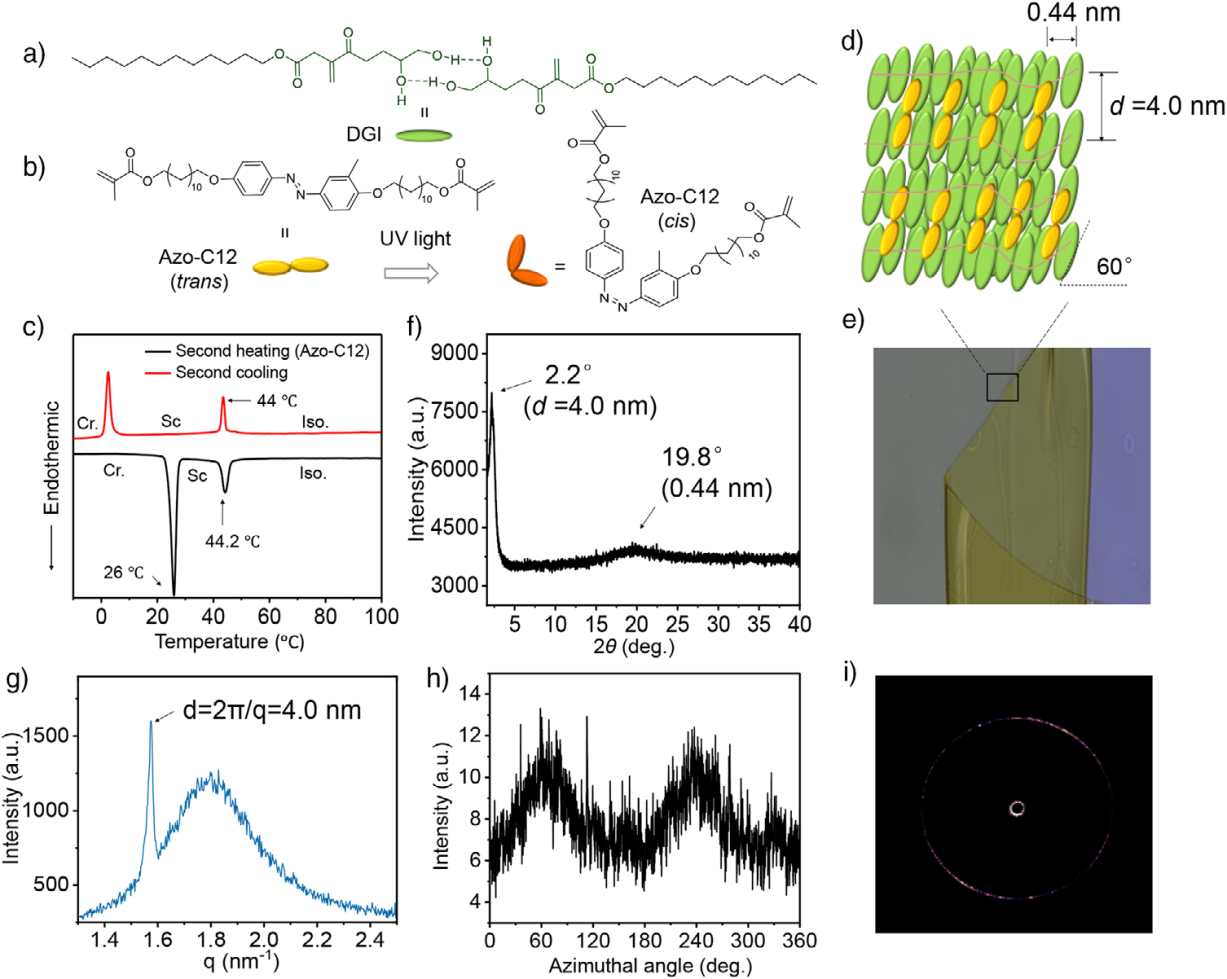
Chemical structures of the monomers and structural characterization of the layered polymer films. a) Chemical structure of a DGI bilayer. DGI monomers contain double bonds that enable polymerization into linear PDGI polymers. Hydrogen bonds can form between the molecules, creating a relatively rigid core structure with flexible long alkyl chain. b) Chemical structure of azo‐C12 monomers and their molecular geometry change from *trans* to *cis* under UV light irradiation. c) DSC thermographs of the azo‐C12 monomers during the second heating and cooling cycle at a constant rate of 5 °C min^−1^. Cr., crystalline; Sc, smectic; Iso., isotropic. d) and e) Schematic illustration of the crosslinked polymer network and a photo of the freshly synthesized film (folded). f) Wide‐angle X‐ray scattering of a freshly prepared polymer film, with the film placed directly onto the testing platform. g) Small‐angle X‐ray scattering of the film. The sharp peak at *q* = 1.57 nm^−1^, corresponding to the interlayer distance (*d*) of ∼4.0 nm. (h) Azimuthal angle plots of the polymer film, integrated over the range of 2*θ* = 2.16°–2.34°, and i) corresponding 2D X‐ray diffraction pattern.

Excellent adhesives typically require the fabrication of soft surfaces with microstructures, such as mushroom‐shaped or pillar‐like structures. In this study, the film structure is simple and does not require complex surface patterns. The films consist of a crosslinked polymer network with two main components. The first component is a polymerizable monomer, dodecyl glyceryl itaconate (DGI), which readily forms a bilayer‐like alignment (Figure 1a). DGI monomers have been previously used to create layered soft materials and polymeric actuators.^[^ ^19^, ^20^, ^21^, ^22^, ^23^, ^24^ ^]^ Due to the long alkyl chain (C12), DGI monomers easily form a bilayer stacking structure via van der Waals interactions. However, their use in adhesion systems has not yet been experimentally demonstrated in the literature. The heads of each DGI molecule contain two hydroxyl (─OH) groups, which are available for hydrogen or adhesive bonding. It has been suggested that there is a correlation between the number of ─OH groups and the strength of adhesion.^[^ ^25^, ^26^ ^]^ Additionally, DGI monomers exhibit no specific absorption in the 300–600 nm range, indicating their high optical transparency (Figure S1).

The second component is a photoresponsive azo‐C12 monomer, designed with two double‐bond termini that act as chemical crosslinkers for synthesizing polymer films (Figure 1b). Azo‐C12 is a novel smectic liquid crystalline (LC) monomer, and smectic LCs are known for their molecularly ordered, layered structure. The long and flexible C12 alky chains with polymerizable terminals that linkage to a rigid azo core structure enhance the formation of smectic phase,^[^ ^27^, ^28^ ^]^ whereas similar azo monomers with shorter alkyl chains (e.g., azo‐C6) do not exhibit this property. Their thermal and optical behavior as well as phase formation were characterized by differential scanning calorimetry (DSC) and temperature‐variable polarized optical microscopy (POM). DSC thermograms show two endothermic peaks at 26.0 °C and 44.2 °C during the second heating process (Figures 1c and S2). POM photographs of azo‐C12 further confirmed the typical fan‐shape textures characteristic of smectic phases upon cooling from the isotropic liquid phase (Figure S3, Movie S1). The polymerization of these LC crosslinkers not only creates layered molecular orientation in the films but also partially inhibited the crystallization of DGI, lowering its crystallization temperature by approximately 8 °C (Figure S4). Photoresponsive smectic LC monomers with low‐temperature phase transitions (<50 °C) that function as chemical crosslinkers for polymer synthesis are rarely reported in the literature.

By mixing these two synthetic monomers in a molecular alignment planar cell (E.H.C., cat. no. KSRP‐50/B107P1NSS) and performing polymerization with a thermal initiator, films with a nanoscale layered structure and covalently crosslinked polymer networks can be synthesized (Figure 1d,e). Wide‐ and small‐angle X‐ray scattering measurements were conducted to characterize the polymer structure. A broad diffraction peak was observed in the wide‐angle region (2*θ* = 19.8°), corresponding to the intermolecular distance of 0.44 nm. In the small‐angle region (2*θ* = 2.2°), a sharp diffraction peak was detected, indicating an interlayer distance of *d* = 4.0 nm (Figure 1f). From the small‐angle X‐ray measurements, a sharp diffraction peak at *q *= 1.57 nm^−1^ and a broad peak at *q *= 1.80 nm^−1^ was observed. The sharp peak corresponds to the previously mentioned interlayer distance of *d* = 4.0 nm (Figure 1g). The broader peak near the sharp peak likely arises from a phase transition of the aged film occurring at a very local length scale. To confirm this, the domain sizes were calculated according to the Scherrer equation, a mathematical formula used to estimate the size of organized regions,^[^ ^29^, ^30^ ^]^

(1) $$D=\frac{K\lambda }{\beta \cos\theta }$$

where *D* is the mean size of the ordered domains, *K* is a dimensionless shape factor (*K *= 0.89), λ is the wavelength of the X‐rays (*λ *= 0.108 nm), β is the width (full width at half‐maximum) of the X‐ray diffraction peak and *θ* is the Bragg angle. The calculated domain structures at *q* = 1.57 nm^−1^ and 1.80 nm^−1^ is 223.6 and 14.5 nm, respectively. These results indicate that the phase transition occurred on a very small scale (spanning only a few molecular lengths) compared to the main domain structure of ordered layers.

The theoretical molecular length (l) of azo‐C12 and bilayer structures of PDGI were evaluated to be 4.71 and 4.60 nm, respectively (Figure S5), and thus d/l∼0.86 implies a tilted structure, yielding a smectic C phase in the polymer film. The tilt angle defined between the direction parallel to layers and the director of molecular alignment was calculated to be 60° from the equation *α* = sin^−1^ (0.86). This is consistence with the results from the orientation characterization of the nanoscale structures from 2D SAXS (Figure 1h,i). As shown in Figure 1h, the azimuthal angle plot features two peaks at 60° and 240°, indicates a well‐assembled molecular orientation in the polymer films. The nanoscale stacked structure of ∼4 nm in length can be considered as oriented molecules for the adhesive films. Additionally, azo‐C12 molecules exhibit thermally stable *trans* state under dark conditions and reversibly change to a more volume‐occupied *cis* state under UV light irradiation.

**Figure 2:**
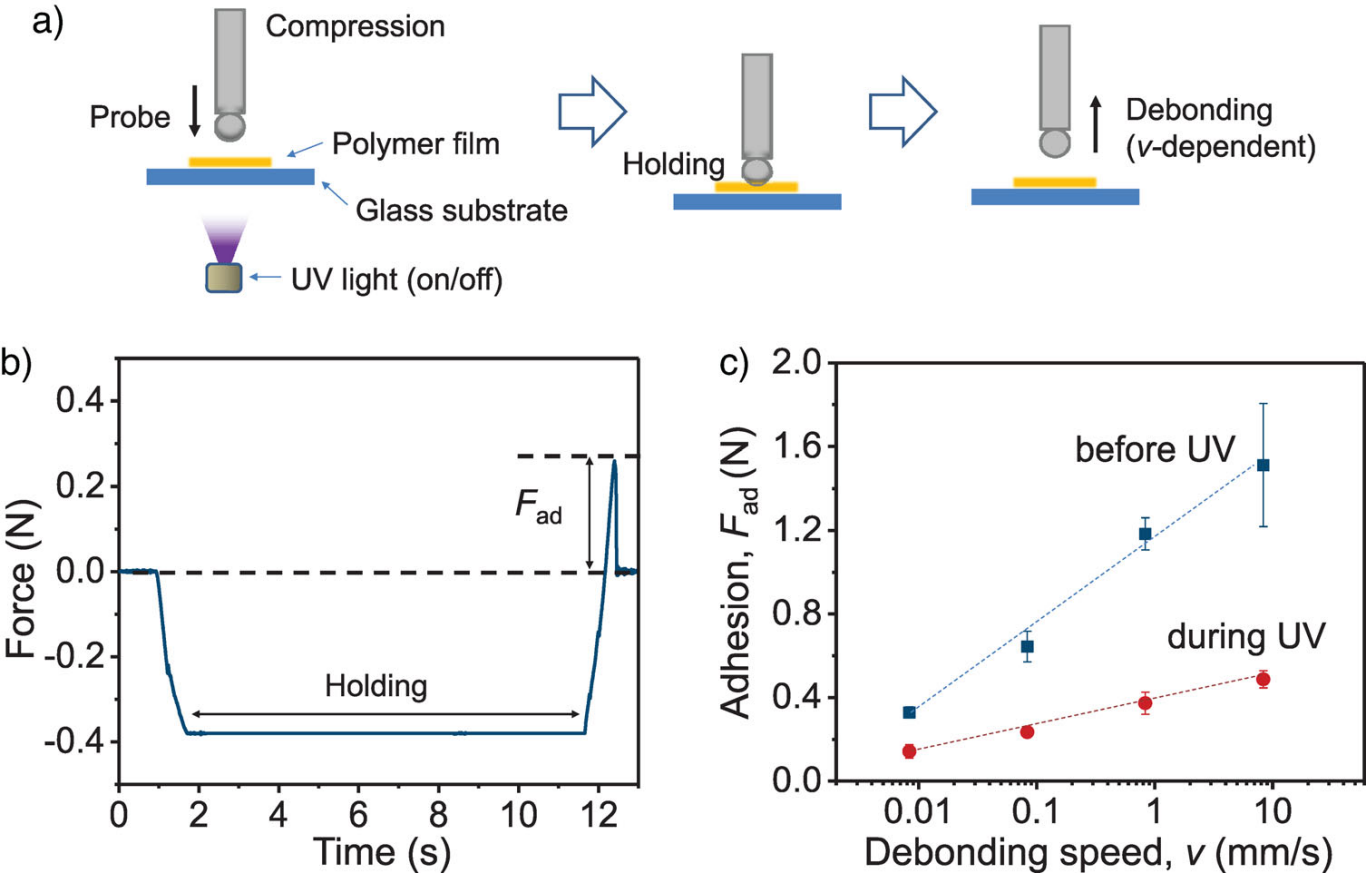
Measurement of adhesive force of the film before and during UV light irradiation. a) Schematic illustration of the experimental setup. b) Time evolution of the forces for three different testing protocols in (a), compression, holding and debonding. c) Adhesion force (*F*_ad_) of the film before and during UV irradiation at different debonding speeds.

An important issue in adhesive films is determining whether or to what extent light stimulation causes the adhesion change in the polymer networks. To measure adhesive force, a film (10 mm × 10 mm × 20 µm) was fixed on a glass slide, and a 10 mm glass sphere connected to a force transducer was brought into contact using a micromanipulator with a preloading force of 0.4 N (Figure 2a). Consequently, the characteristic force–time data for the adhesive contact between the spherical probe and the sample were obtained (Figure 2b). After maintaining a constant normal force for a set duration, the probe was pulled vertically at varying debonding speeds to measure adhesion force. To assess UV‐induced adhesion changes, the probes was similarly pressed onto the film, held for 10 s and then detached at a constant speed under UV irradiation. The adhesion forces before and during UV exposure were measured (Figure 2c). The results showed that the adhesion force averaged 1.18 N without UV, whereas under UV, it decreased to 0.37 N at a pulling speed of 1.0 mm s^−1^. When the UV light was turned off, adhesion recovered without additional light exposure. We also investigated whether the speed of controlled peeling could be used to adjust the adhesion force. As shown in Figure 2c, the film exhibits an increase in adhesion force with increasing debonding speeds (*v*) either before or during UV irradiation.

The films exhibit velocity‐dependent adhesion force because the detachment process is related to the interfacial fracture energy and the viscoelastic properties of the polymer films.^[^ ^31^ ^]^

(2) $$\Gamma \left(v\right)=\Gamma_{0}\left(1+\varphi \left(a_{T}\nu \right)\right)$$

where *Γ*_0_ is the threshold adhesion energy for zero crack propagation velocity between the polymers and the contacted probe and φ(*a_T_* ν) is a velocity‐dependent dissipative factor related to the loss in viscoelastic energy during the interfacial crack propagation at speed *ν*. φ(*a_T_* ν) should be related to the bulk viscoelastic characteristics of the polymeric films.

**Figure 3:**
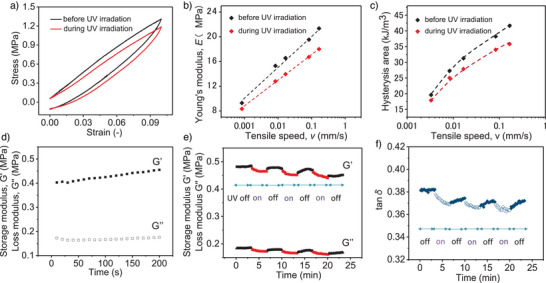
Photoswitchable mechanical properties of the polymer films. a) Stress–strain curves of the free‐standing film under elongation before and after UV irradiation at a tensile speed of 0.5 mm min^−1^. b) Young's modulus and c) hysteresis area of the film before and during UV irradiation at varying tensile speeds. d) The evolution of the storage modulus (G′) and loss modulus (G″) versus time without UV irradiation. e) The photoswitchable behavior of the storage modulus, loss modulus, and f) tan δ of the film under UV light on/off switching.

To further investigate this, we measured the mechanical properties of the films before and during light irradiation. As shown in Figure 3, tensile stress–strain elongation and rheometer were used to characterize the photoswitchable mechanical behaviors of these films. The tensile elongation and relaxation tests revealed a noticeable hysteresis during elongation and relaxation, indicating energy dissipation during mechanical elongation in the direction parallel to the molecular alignment (Figure 3a). During UV irradiation, the hysteresis area (energy dissipated during elongation) of the same sample in the same setting is reduced due to the presence of light‐responsive azo chromophores (Figure 3a). This phenomenon was also observed at other tensile speeds, suggesting that the polymer films exhibit photoswitchable Young's modulus and energy dissipation (Figures 3b,c, and S6).

The effect of light irradiation on the viscoelastic behavior of polymer films was investigated using an amplitude oscillatory shear rheometer. The UV light was integrated into the rheometer and programmed for automatic control with a predetermined switching time (Figure S7). UV on/off switching was performed to elucidate the temporal characteristics of the photoresponsive viscoelastic behavior of the films. Figure 3d shows the time sweeps of storage modulus (G′) and loss modulus (G″) at a small strain of 0.5%, suggesting no significant change in the modulus without UV irradiation. Then, the variations of G′, G″, and loss factor (tan δ) during UV‐ light on/off switching were investigated. When UV light was turned on, the modulus responded immediately. The average storage modulus decreased from 0.48 to 0.46 MPa, the loss modulus decreased from 0.18 to 0.17 MPa (Figure 3e). Upon UV irradiation, the films quickly returned to their initial state. Thus, tan δ (calculated from the ratio of G″ to G′) varies between high and low values with UV on/off cycling, indicating that the viscoelasticity of the film undergoes a rapid and reversible change from a more viscous to a more elastic state under UV irradiation (Figure 3f). The changes in viscoelasticity of the polymer films contributed to the observed adhesion changes of the films before and during UV irradiation. However, the light‐induced changes in viscoelasticity are not as large, and this may be a secondary rather than a direct factor for the rapid adhesion/detachment that occurs in these films. With such consideration, other properties of the films before and during light irradiation were investigated.

As aforementioned, insects, such as *Oecophylla smaragdina*, control attachment and detachment quickly by adjusting their adhesive contact area through leg movements. When walking upside down on a smooth substrate, they only use 14% of their pad's maximum contact area, but when carrying a 30 mg load (approximately six times their body weight), they required a larger contact area (approximately 60%).^[^ ^32^ ^]^ Thus, to achieve fast detachment, external stimuli should trigger shape changes in the toes to quickly reduce the contact area, thereby decreasing the adhesive fracture energy threshold. The orientation of nanofibers on adhesive pads is critical for detachment as properly aligned nanofibers minimize contact area and reduce the force required for peeling above a critical angle from the substrate.^[^ ^3^ ^]^

**Figure 4:**
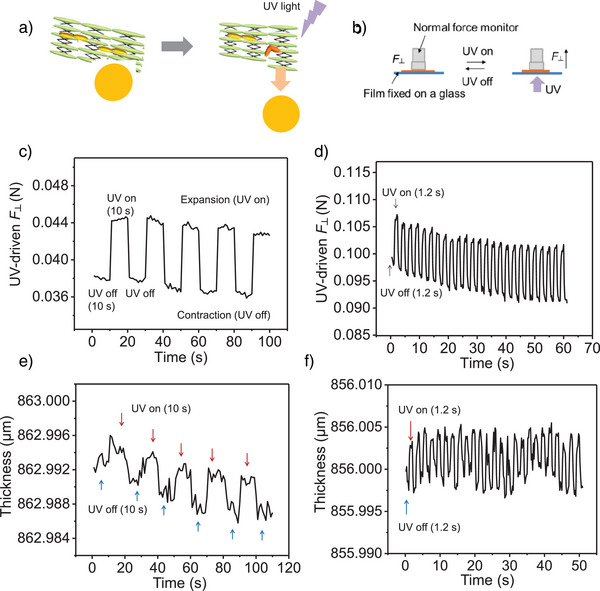
Light‐driven abrupt and reversable shape change in the polymer film. a) Detachment of an adhered ball from the film due to light‐induced expansion. b) Measurement of UV‐driven normal force (*F*_⊥_) during UV on/off switching, where *F*_⊥_ is force perpendicular to the molecular orientation. c) and d) Time dependence of *F*_⊥_ in the film under 365 nm UV for (c) 10 s and (d) 1.2 s. e) and f) Thickness change of the film during UV on/off switching. Total thickness includes the glass substrate, with *F*_⊥_ controlled at 0.001 N during measurement.

Inspired by this biomimetic detachment, films can be designed to exhibit rapid detachment through an abrupt and reversable shape change upon UV light exposure. As shown in Figure 4a, an adherend (e.g., ball) was attached on a film fixed on a glass substrate. When UV light (365 nm) was focused on the film, the ball detached due to the increase in the local film thickness. The thickness increase generated a normal force (*F*_⊥_) in the direction perpendicular to the molecular alignment when the gap between force monitor and substrate was minimized (Figure 4b). It was found that *F*_⊥_ increased by ∼0.006 N and remained at that value and then immediately decreased when UV light was off (Figure 4c). The film underwent rapid expansion and contraction (shrinking), repeating over 50 times within 60 s by switching the UV light on and off (Figure 4d). The shape change of the film is also characterized by the variation in thickness during light on/off switching (Figure 4e,f). When the UV light is turned on, the film thickness increases, and when the light is off, the thickness decreases. The change in thickness ranges between 0.004–0.005 µm with *F*_⊥_ controlled at 0.001 N. Thus, the thickness change aligns with the normal force analysis, both confirming expansion under UV irradiation and contraction when the light is off. As shown in Figure 4f, both expansion and recovery occur rapidly, with light switching time taking around 1 s. Additionally, the sample remained stable after over 2000 cycles (Figure S8), demonstrating high durability. Thus, high‐speed mechanical motion (expansion and shrinking) of the film was achieved for thousands of cycles through UV on/off switching.

When the majority of azobenzene units in the film are in the *trans* state, the film thickness is at its minimum. UV irradiation increases the *cis* fraction, causing film expansion and normal force to rise initially. However, under typical laboratory conditions, the tested films usually contain a certain fraction of *cis* isomers. Short‐time UV irradiation quickly increases the *cis* fraction, causing the film thickness and normal force to reach their maximum (Figure 4d,e). When the UV light is turned off, the film undergoes rapid shrinking. However, since many *cis* isomers do not quickly revert to the *trans* state, the film remains in a mixed *cis‐trans* state, leading to intermediate normal force and thickness values. The successive decrease in both parameters over long time switching (Figure 4d,e) is attributed to the ongoing *cis‐to‐trans* thermal relaxation, which gradually restores the polymer network toward its thermally stable *trans* conformation.

In nature, despite long years of studies on animal adhesion systems (e.g., insects, spiders, and geckos), the mechanisms of rapid attachment during walking remain controversial.^[^ ^33^ ^]^ Different hypotheses have been proposed to explain the adhesion mechanisms, such as sticking fluid, microsuckers, and electrostatic forces.^[^ ^34^, ^35^ ^]^ Recently, strong evidence have suggested that adhesion of gecko setae is caused by van der Waals forces.^[^ ^36^ ^]^ In this study, the films consist of stacked PDGI, containing ─OH groups that enhance intermolecular interactions with adherents. Additionally, the flexible C12 alkyl chains in PDGI promote chain mobility, enabling rapid stress relaxation and allowing the polymer to quickly adapt to changes in light exposure. The azo‐C12 molecules, also featuring long alkane chains, contribute to films with high free volume, while their LC “fluid” nature and long‐range smectic order enhance interface compliance and UV‐driven motion in the polymer networks. To investigate the effect of alkane chains on the azobenzene unit, we synthesized azobenzene molecules with a different substituent (azo‐C8) and incorporated them into polymer films. However, the films crosslinked by azo‐C8 exhibited significantly lower adhesion compared to C12‐based films (Figure S9). These results highlight the crucial role of substituents in azo unit in influencing both the molecular properties and the adhesion performance of the polymers.

**Figure 5:**
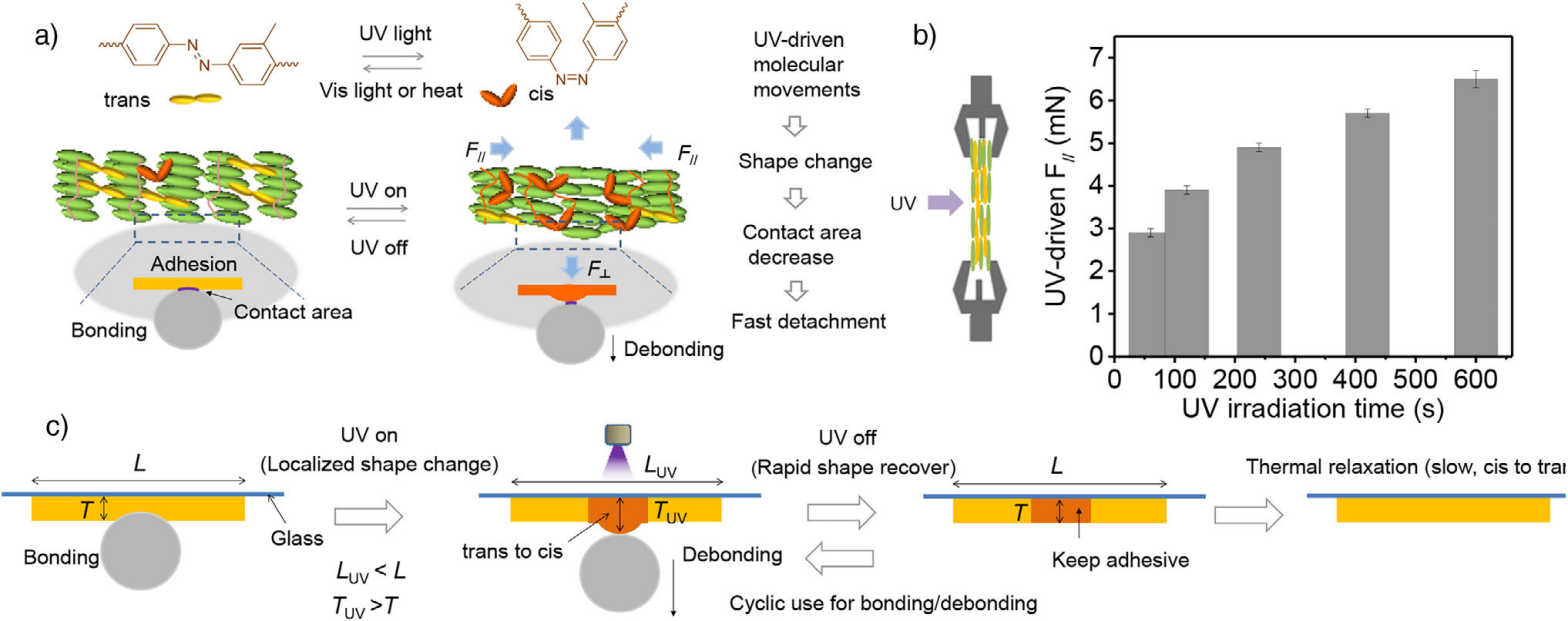
Working principle of fast and reversable adhesion/detachment in the polymer film. a) UV light induces molecular changes in azo crosslinkers, causing polymer network deformation and generating internal forces. The expansion (perpendicular to molecular alignment) reduces the contact area with the ball, leading to its detachment upon UV irradiation. *F*_//_: force parallel to the molecular alignment; *F*_⊥_: force perpendicular to the molecular alignment. b) Measurement of the force in the direction to parallel to the molecular alignment upon UV irradiation. c) Schematic illustration of the working principles for the fast and reversable adhesion/detachment. *L*: length of the film; *L*_UV_: length of the film during UV irradiation; *T*: thickness of the film; *T*_UV_: thickness of the film during UV irradiation.

In a freshly prepared film, most azo chromophores are in the thermally stable *trans* conformation, while the PDGI adopts a layered structure chemically crosslinked by the azo molecules (Figure 5a, left). Upon UV light exposure, the azo molecules switch to the *cis* conformation, activating polymer chains movements.^[^ ^24^, ^37^ ^]^ The UV‐driven polymer network movements cause the film to locally expand (thickness increase) onto the surface of the ball, reducing contact area and detaching the ball (Figure 5a, right). Additionally, the light‐induced shape change generates forces either parallel (*F*_//_) or perpendicular (*F*_⊥_) to the PDGI and azo mesogen alignment. We also measured the UV‐driven force in the direction parallel to the molecular alignment (Figure 5b). The force in this direction increased negatively with UV irradiation time, indicating a decrease in length parallel to molecular alignment (Figure 5b). To maintain volume, expansion in the direction perpendicular to the molecular alignment becomes obvious during UV exposure. In situ SAXS measurements of the film during UV irradiation revealed some changes in the molecular alignment. Specifically, the broad shoulder peak clearly shifted to higher *q*‐values, indicating that nanostructures with lengths less than 3.5 nm gradually increased due to bent molecular geometry of azo molecules upon UV irradiation (Figure S10). This change partially disrupted the originally layered structure.

During UV irradiation, the light is localized on the ball, with increased irradiation on the contacted area due to reflection from the ball, leading to thickness expansion, particularly at the contact area. This abrupt shape change reduced the contact area between the attached ball and the film and consequently decrease the adhesive force, resulting in quick debonding (Figure 5c). Once the light is turned off, the films return to their original shapes immediately. Although hysteresis occurs when the film is extensively stretched along the molecular alignment direction, small strain variations perpendicular to it recover rapidly. Notably, the response time is quick; even with only 1.2 s of irradiation, the film expands in thickness and immediately returns to its original state when UV is switched off. This working principle is similar to the use of mechanical stimuli by insects to reduce contact area, which is conceptually different from other adhesion systems reported that utilize light‐induced solid‐to‐liquid phase transitions, light‐thermal induced phase transitions or light‐controlled surface topographical change (with worm‐like fingerprint textures).^[^ ^38^ ^]^

The effects of irradiation light intensity and film thickness on light‐triggered adhesion performance were investigated (Figures S11, S12). Increasing the light intensity from 0 to 120 mW cm^−^^2^ gradually resulted in a decrease in adhesion force. Additionally, thicker films (20 and 40 µm) exhibited higher adhesion compared to thinner films (6 and 10 µm), likely due to improved surface compliance and increased contact area. These results highlight the importance of optimizing both light intensity and film thickness to achieve an optimal balance in adhesion performance. The impact of crosslinking density on the light‐responsive behavior was also investigated by varying the azo‐C12 content. Films with lower azo‐C12 content (4.2 and 11.5 wt%) were unable to form free‐standing films. The results demonstrate that adhesion force decreased with increasing crosslinker content (Figure S13). This is attributed to the dense network formed by higher crosslinking, which restricts polymer chain mobility. As adhesion is often dependent on surface adaptability, reduced chain mobility weakens the film's ability to conform to surfaces, resulting in a decrease in adhesion.

UV light irradiation triggers *trans‐to‐cis* isomerization, which generally disrupts the ordered mesogen alignment because the *cis* state demonstrates a non‐mesogenic state owing to its bent molecular geometry.^[^ ^15^ ^]^ When the UV light was turned off, azo molecules try to return to their thermodynamically stable *trans*‐form. However, the thermal *cis*‐*trans* relaxation is long (several hours/days) in the film state, although they are reversible and can undergo hundreds of successive irradiation cycles.^[^ ^39^, ^40^ ^]^ We measured the lifetime of the *cis* state in these films, and the results confirmed that at least several hours were required for the *cis*‐rich film to fully return to its *trans* state (Figure S14). However, this slow *cis‐to‐trans* thermal relaxation did not affect the films' quick return to their original shape due to the rapid stress relaxation of PDGI. This property makes it conceptually distinct from other photoresponsive adhesion systems, which typically require long processing times and two light stimuli, one triggering detachment and the other light irradiation restoring adhesion.^[^ ^41^ ^]^ The long alkyl chains of both monomers and LC phase of azo crosslinkers also enable a sufficient free volume of the film and a good balance of softness and viscoelasticity. Thus, the two components in the films can achieve ideal performances for both adhesion and detachment using a simple material design.

**Figure 6:**
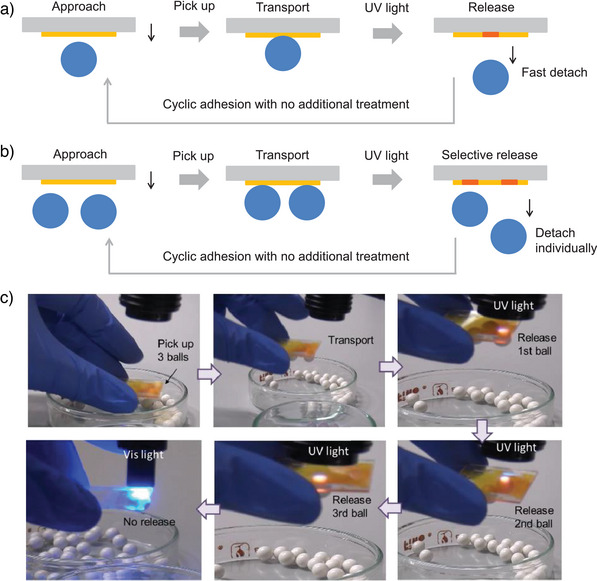
Schematic of the polymer film used for adhesion/detachment of a) a polystyrene ball and b) several balls and their subsequent detachment individually using UV light. c) Photographs of real pickup–delivery–release cycles of adhesion/detachment, showing selective response to UV light (365 nm) and no similar response to visible light (465 nm).

We conducted adhesion and release experiments to demonstrate the switchable adhesion of these dynamic responsive films (Movie S2). In the first experiment, a 6 mm polystyrene ball (0.2 g) was attached to the coated polymer film and detached upon UV irradiation. As shown in Figure 6a and Movie S2, when the coated surface approached the plastic ball, pressure was applied to attach the ball to the coated film. After transporting the ball, UV light (365 nm, ∼100 mW cm^−2^) was applied for 1–2 s to detach the ball. The light intensity dropped to 80% after passing through the glass, and to 22 mW cm^−2^ after passing through the film. Interestingly, the UV‐driven force causes the ball to fall at high speed. Since the film surface remains adhesive, it can be immediately used for the next cycle of bonding/debonding without the need for additional treatments (such as visible light) or waiting time. This pickup–delivery–release working cycle can be repeated many times without degradation. Moreover, this polymer coating allows the transport of several objects simultaneously and the sequential release of these balls individually by using UV light (Figure 6b,c, and Movie S3). We also irradiated the film with another visible light source (465 nm), but it did not effectively cause the balls to detach (Movie S4). Regarding the photothermal effect, although it cannot be completely ruled out that photogenerated heat contributes to molecular movement, the very short‐time UV light (100 mW cm^−2^) exposure can hardly cause a noticeable increase in the surface temperature of the film (Figure S15).

The free‐standing films were cut into a strip (1 mm wide) and clamped vertically at both ends on a tensile machine. Subsequently, objects of different materials and shapes were prepared to test their adhesion (Figure S16a). These objects included cylindrical polystyrene, silicon wafers, fluorine rubber tape, polypropylene pipette tips, and metal aluminum, all of which adhered to the film, even though some of these items were hundreds of times heavier than the film (Figure S16a). These experiments demonstrated the film's clean, strong adhesion to flat, spherical, or convex surfaces of different materials, indicating their interface compliance and conformability to surfaces with a variety of roughness and toughness.

Furthermore, adhesion in high‐humidity or underwater environments is important for adhesive materials. Achieving strong adhesion in water using supramolecular adhesives has been challenging due to water‐induced instability.^[^ ^42^, ^43^ ^]^ As shown in Figure S16b and Movie S5, the freestanding polymer film function as an adhesive in underwater and ethanol environments. These chemically crosslinked polymer networks provide stability as non‐crosslinked polymers are prone to dissolution in organic solvents. This adaptability expands the film's potential applications across various environments. While the film demonstrates adhesion to solid surfaces, it also exhibits antifouling properties, preventing unwanted contamination from aqueous or oil‐based substances (Figure S17). This combination ensures reliable adhesion while maintaining surface cleanliness, making it suitable for repeated use.

Light‐induced adhesion/detachment mechanisms have been reported in the literature, including light‐thermal‐effect‐controlled phase changes, viscoelastic changes, photoisomerization‐controlled viscoelasticity, and light‐controlled surface topographical changes. However, these proposed mechanisms are difficult to explain the fast, reversible, and residual‐free adhesion/detachment observed in this system. The shortest reported (detaching) time in literature is ∼5 s in the systems using light‐controlled surface topographical change.^[^ ^38^, ^44^ ^]^ There is also an interesting demonstration of three‐layered structured adhesive films with mushroom‐shaped surface structure that detach objects in 5 s under a light intensity of 500 mW cm^−2^.^[^ ^15^ ^]^ Thus, producing a fast adhesion/detachment that can be retained many cycles is still difficult.^[^ ^45^ ^]^

For example, photoswitchable materials with adjustable moduli have been reported as suitable candidates for switchable adhesives. By adjusting the modulus using two types of light (UV and visible), the adhesive can be turned into high or low adhesion strengths.^[^ ^46^ ^]^ In addition, phase‐change materials can be transformed from solids to liquids and are ideal switchable adhesives.^[^ ^12^, ^47^, ^48^ ^]^ However, achieving a fast and clean detachment using these systems is difficult. Furthermore, light‐responsive topographical deformations have been proposed for use in adhesive polymer systems. However, it requires an additional visible light (e.g., blue light) to recover the adhesion ability for the next adhesion/detach cycles and also need relatively long irradiation time to detach the objects, limiting the continued pause‐free working cycles.^[^ ^44^ ^]^ Therefore, more desirable materials need to be developed for rapid adhesion/detachment based on a different working mechanism.

**Figure 7:**
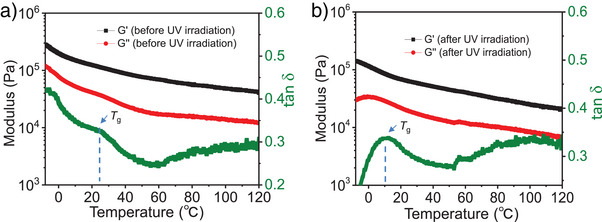
Temperature dependence of viscoelastic parameters (G′, G″, and tan δ) of the polymer films a) before and b) after sufficient UV light irradiation.

As shown in Figure 7, the temperature‐dependent rheological behavior of the polymer film, including the G′, G″, and tan δ, was investigated under a 0.5% strain at a 1 Hz frequency in the dark. The glass transition temperature (*T*_g_) of the polymer, defined as the temperature at which tan δ reaches its maximum, was approximately 25 °C before UV light irradiation and decreased to approximately 10 °C after UV light irradiation (Figure 7a,b). Upon UV irradiation, the polymer chains absorb photons, activating their dynamic motions. First, UV‐induced *trans‐to‐cis* isomerization of the azo units increases free volume and enhances chain mobility, lowering the temperature at which the polymer transitions from a glassy to a rubbery state, thereby decreasing *T*_g_. Second, both G′ and G″ decrease, indicating overall softening of the polymer network. Third, while increased molecular mobility typically leads to a higher tan δ, the disruption of the layered structure in this system weakens intermolecular interactions and reduces internal friction, resulting in lower energy dissipation and thus a decrease in tan δ.

A significant mismatch exists between timescales of photoisomerization and adhesion switching. The slow *cis‐to‐trans* thermal relaxation complicates the explanation of the rapid attachment/detachment observed under UV light alone. Therefore, the mechanism needs to be explained in terms of polymer dynamics rather than simply the *trans* or *cis* states of the polymers. Notably, the film maintain adhesion and are ready for subsequent attachment/detachment, regardless of their predominance in the *trans* or *cis* states. Additionally, in this crosslinked film, even a small molecular change (from *trans* to *cis*) effectively activates localized movements within the polymer network. For instance, after 2 s of UV irradiation at an intensity of 41 mW cm^−2^, approximately 3.4% of the covalently embedded azo molecules in the film undergo conversion from *trans* to *cis* isomers (Figure S18). Although the *cis* isomer constitutes only 0.68 wt% of the film's total weight (as the total azo content being 20 wt%), this small fraction is sufficient to activate mechanical motion of the polymer network and detach objects. Due to the crosslinked nature of the polymer network, the elastic energy accumulated during UV‐driven shape changes must be released, ensuring the system is rapidly ready for the next bonding cycle without requiring additional external stimuli.

## Conclusion

We have developed a polymer film with a nanoscale layered structure that enables high‐speed mechanical motion switching (expansion and contraction) under controlled UV irradiation. The film undergoes rapid (∼1 s response time) and reversible shape changes with UV on/off switching, maintaining stability over 2000 cycles. These chemically crosslinked films provide adhesive/detachment capabilities triggered by UV light, with fast response and high reproducibility. Driven by light‐induced motion of the polymer network and photoswitchable *T*_g_, the films can release objects upon UV exposure and regain adhesive properties immediately without additional stimuli. These soft and flexible films are well‐suited for light‐controlled pickup–delivery–release systems, enabling the transport of solid objects with diverse materials and shapes. They show promise as reusable adhesives in everyday products such as tapes, fasteners, and toys, as well as in advanced technologies, including microelectronics (e.g., temporary adhesives for semiconductor manufacturing) and potential space applications.

## Supporting Information

The authors have cited additional references within the Supporting Information.^[^ ^49^, ^50^, ^51^, ^52^ ^]^

## Conflict of Interests

The authors declare no conflict of interest.

## Supporting information



Supporting Information

Supporting Movie S1

Supporting Movie S2

Supporting Movie S3

Supporting Movie S4

Supporting Movie S5

## Data Availability

The data that support the findings of this study are available from the corresponding author upon reasonable request.
