# Supplementary material for: Biomimetic Adhesion/Detachment Using Layered Polymers with Light‐Induced Rapid Shape Changes
Source: Angew Chem Int Ed Engl. 2025 Apr 4;64(23):e202503748. doi: 10.1002/anie.202503748 (PMC12124347; doi:10.1002/anie.202503748)
Supplement: Supplementary file 1 — Supporting Information [file ANIE-64-e202503748-s005.docx]

Supplementary Materials for

**Biomimetic Adhesion/Detachment using Layered Polymers with Light-induced Rapid Shape Changes**

*Youfeng Yue^1, *^, Yasuo Norikane^1^, Eiji Nishibori^2^*

*Corresponding author. Email: [yue-yf@aist.go.jp](mailto:yue-yf@aist.go.jp)

**This file includes:**

Supplementary Text

Figure S1 to S18

**Other Supplementary Materials for this manuscript include the following:**

Movies S1 to S5

**Supplementary text**

**Materials**

Tetrahydrofuran (dehydrated), toluene (super dehydrated), N, N-dimethylformamide, hexane, ethyl acetate, and hydrochloric acid were purchased from Wako Pure Chemical Industries, Japan. Dimethyl sulfoxide-d6 (DMSO-d6), and deuterated chloroform, (CDCl_3_) were purchased from Aldrich. Itaconic acid anhydride, dodecanol, pyridinium p-toluenesulfonate, glycidol, sodium nitrite, HO(CH_2_)_12_Br, K_2_CO_3_, KI and methacryloyl chloride were purchased from TCI chemicals, Japan and used as received. 1,1’-azobis(cyclohexane-1-carbonitrile) was purchased from Wako Pure Chemical Industries Ltd., Japan, and used as an initiator for polymerization. The thin slide glass (Matsunami Glass Ind., Ltd. Square microscope cover glass No.1, 18 mm × 18 mm or 22 mm× 22 mm, thickness: 0.12-0.17 mm), were used as the substrates for optical observations.

**Synthesis of dodecyl glyceryl itaconate**

Dodecyl glyceryl itaconate (DGI) was synthesized using a modified method reported by Tsujii et al.^[49]^ Specifically, itaconic acid anhydride was reacted with dodecanol at 100 °C for 2 hours. After the reaction, a large amount of hexane was added to the mixture under stirring, causing the white-colored product (dodecyl itaconate) to precipitate. The crude products were recrystallized from ethanol. The product was then reacted with glycidol at 100°C in toluene (super dehydrated) using pyridinium *p*-toluenesulfonate as a catalyst (under N_2_ atmosphere). The products were purified using silica gel column chromatography (hexane/ethyl acetate= 4:6), and the pure white compounds were recrystallized from an acetone/hexane mixture.

**Synthesis of azo-C12 smectic LC monomers**

*Synthesis of 4,4’-Dihydroxy-3-methlazobenzene*

4,4’-Dihydroxy-3-methlazobenzene was synthesized using a standard two-step azo-coupling reaction. 4-aminiphenol (4.36 g) was dissolved in a hydrochloric acid solution (2.4 mol/L, 50 ml) and the solution was kept at -3 °C. Sodium nitrite (3.32 g) in water (4 ml) was added dropwise to generate the diazonium salt. The mixture was stirred for 30 min and then added dropwise to a solution of o-cresol (4.32 g) in sodium hydroxide solution (20%, 16 mL) at -3 °C, resulting in precipitation of yellow solids. The reaction mixture was stirred at room temperature for ~20 hours. Then diluted hydrochloric acid was added to neutralize the mixture. The brown solids were filtered, washed with water. The pure compound (4,4’-dihydroxy-3-methlazobenzene) was purified by column chromatography (ethyl acetate/hexane=1:2) and recrystallized from acetone/hexane mixture. ^1^H NMR (400 MHz, DMSO-d6) δ 10.08 (s, 1H), 10.04 (s, 1H), 7.70 (d-d, J1 = 6.8 Hz, J2 = 1.9 Hz, 2H), 7.60 (d, J= 2.0 Hz, 1H), 7.55 (d-d, J1 = 8.4 Hz, J2 = 2.4 Hz, 1H), 6.92 (d, J = 8.4 Hz, 1H), 6.90 (d-d, J1 =6.8 Hz, J2 = 1.9 Hz, 2H), 2.20 (s, 3H) ; ^13^C NMR (125 MHz, DMSO-d6) δ 160.1, 158.4, 145.5, 145.3, 125.1, 124.3, 122.6, 116.0, 115.1, 16.2.

*Synthesis of 12-(4-((4-((12-hydroxydodecyl)oxy)-3-methylphenyl)diazenyl)phenoxy)dodecan-1-ol*


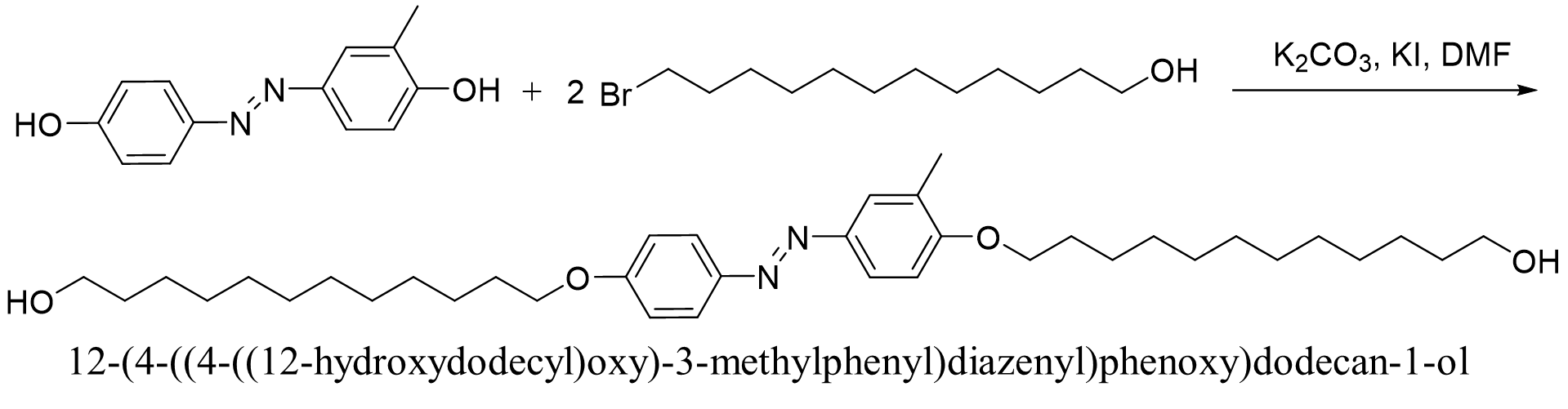


4,4’-Dihydroxy-3-methlazobenzene (0.767 g), 1.39 g K_2_CO_3_, and 0.005 g KI was dissolved in 40 ml N, N-dimethylformamide, and a solution of HO(CH_2_)_12_Br (2.23 g) in 25 ml DMF was added. The mixture was stirred at 120 °C for ~70 h. After the reaction, a large amount of water was added to the mixture to precipitate the products. The crude products were filled off and recrystallized from ethanol to give a yellow powder. ^1^H NMR (500 MHz, CDCl_3_) δ 7.85 (d, J = 8.9, 2 H), 7.71-7.74 (m, 2 H), 6.98 (d, J = 9.0, 2 H), 6.90 (d, J = 9.3, 1 H), 4.01-4.06 (m, 4 H), 3.64 (t, J = 6.7, 4 H), 2.29 (s, 3 H), 1.78-1.86 (m, 4 H), 1.54-1.59 (m, 4 H), 1.44-1.52 (m, 4 H), 1.26-1.40 (m, 36 H); ^13^C NMR (100 MHz, CDCl_3_) δ 161.04, 159.45, 146.99, 146.37, 127.51, 124.19, 123. 45, 123.38, 114. 64, 110.51, 68.31, 68.25, 63.10, 32.81, 29.66, 29.60, 29.44, 29.39, 29.25, 29.22, 26.11, 26.02, 25.74, 16.41

*Synthesis of 12-(4-((4-((12-(methacryloyloxy)dodecyl)oxy)-3-methylphenyl)diazenyl)phenoxy) dodecyl methacrylate* (Azo-C12)

12-(4-((4-((12-hydroxydodecyl)oxy)-3-methylphenyl)diazenyl)phenoxy)dodecan-1-ol (0.554 g), triethylamine (0.1 g), 4-Dimethylaminopyridine (0.028 g) were dissolved in dehydrated tetrahydrofuran (13.0 mL) at 0 °C with stirring. Methacryloyl chloride (0.39 g) dissolved in dehydrated tetrahydrofuran (3.0 mL) was then added to the solution at 0 °C. The reaction progress was tracked by TLC until most of starting compound was reacted. The reaction was quenched by adding water to the mixture. The products were extracted with chloroform, and the chloroform layer was dried over MgSO_4_. After the solvent was removed, the resulting yellow solid compounds (Azo-C12) were purified by silica gel column chromatography (hexane/ethyl acetate =10:1). ^1^H NMR (500 MHz, CDCl_3_) δ 7.86 (d, J = 9.1, 2 H), 7.75 (m, 2 H), 7.00 (d, J = 9.0, 2 H), 6.91 (d, J = 9.5, 1 H), 6.10 (s, 2 H), 5.56 (s, 2 H), 4.16 (t, J = 6.7, 4 H), 4.03-4.07 (m, 4 H), 2.31 (s, 3 H), 1.96 (s, 6 H), 1.82-1.86 (m, 4 H), 1.66-1.70 (m, 4 H), 1.31-1.58 (m, 32 H); ^13^C NMR (125 MHz, CDCl_3_) δ 167.55, 161.08, 159.47, 147.08, 146.48, 136.62, 127.52, 125.07, 124.22, 123.54, 123.36, 114.69. 110.59, 68.34, 68.29, 64.84, 29.55, 29.52, 29.39, 29.26, 28.65, 26.13, 26.04, 26.00, 18.32, 16.37. HRMS (EI): m/z calculated for C_45_H_68_N_2_O_6_, 732.508; found: 732.509. Melting point: 59-60 °C.

**Supplementary Methods**

**Film fabrication**: The polymer films were made by mixing two synthesized monomers (azo-C12 and DGI) and free radical polymerization of these monomers with a thermal initiator. Specifically, 22 mg DGI, 6 mg azo-C12 monomers, and 0.5 mg initiator were melted by heating at a temperature of 90 ºC in a vial. A small amount of toluene (20 μL) was added to reduce the viscosity of the mixture, which was drawn by capillary pressure into parallel setup containing molecule alignment cells (E.H.C., cat. no. KSRP-50/B107P1NSS). For the polymerization reaction, the sample was heated on a hot plate to 90 °C and maintained for 1.5 hours, followed by an increase in temperature to 120 °C to polymerize the DGI and azo monomers. To synthesize films of different thicknesses, cells with different spacings were used. To synthesize films with different crosslinking densities, the content of azo-C12 crosslinkers was adjusted. The polymerization process was carried out in dark.

**^1^H NMR or ^13^C NMR spectra**: The nuclear magnetic resonance characterization was carried out on a Bruker Avance NMR spectrometer (Switzerland). Multiplicities are abbreviated as follows: singlet (s), doublet (d), triplet (t), and multiple (m). The residual protonated solvent signals were used as internal references for ^1^H NMR and ^13^C NMR spectra (^1^H NMR: δ (CDCl_3_). 7.26 ppm, δ (DMSO-d6). 2.50 ppm and ^13^C NMR: δ (CDCl_3_). 77.16 ppm, δ (DMSO-d6) 39.50 ppm. Multiplicities are abbreviated as follows: singlet (s), doublet (d), triplet (t), and multiple (m).

**Polarized optical microscopy**: The images were taken using an Olympus BX51 polarized optical microscope fitted with a digital camera, and a Linkam 10033 heating/cooling stage. A small amount of azo-C12 monomers was placed on the slide glass (square microscope cover glass No.1, 18 mm × 18 mm). The LC phase patterns of the monomers were observed using the heating/cooling process. The melting points observed from the POM are well consistent with the measurement from differential scanning calorimetry measurements.

**Light sources and intensity measurement**: The light sources used were LED lamps (CCS, HLV-24VV365-4W PCLTL and HLV-24UV365-4WNRBTNJ for λ = 365nm; HLV2-22βL-3W for λ = 465nm). The light intensity was monitored by a Newport 843-R optical power meter with 818-ST2-UV/DB power detector.

**Differential scanning calorimetry**: The phase transition temperatures of the smectic LC azo-C12 monomers were determined using differential scanning calorimetry (DSC, SII Nanotechnology DSC6100). The heating and cooling rates were set to 5 °C /min with N_2_ gas flow 50 ml min^-1^. The corresponding mesophases of azo-C12 monomers were identified by observing their textures using polarized optical microscopy.

**In-situ small-angle X-ray characterization (In-situ SAXS)**: In-situ synchrotron radiation small angle X-ray scattering under UV light irradiation experiments were carried out at SPring-8 BL02B2 beamline.^[50]^ The MYTHEN detector, equipped on the long arm with a length of 1146 mm, was used for the in situ experiment. The in-situ diffraction data were collected with an exposure time of 1 s. The wavelength of incident X-ray was 1.08 Å calibrated using the NIST CeO_2_ standard powder.

**Measurement of the azimuthal angle**: Azimuthal angle (φ) plots of the polymer film were performed on the top of the samples using a Rigaku NANO-Viewer setup with Cu Kα radiation (40 kV and 30 mA, λ = 0.154 nm, camera length = 680 mm, detector: HyPix-3000) at room temperature.

**Tensile tests**: The tensile tests of the films were conducted on a tensile machine (EZ-LX, SHIMATSU) at room temperature with different elongation velocities. The stress (engineering stress) was recorded as the load divided by the original cross-sectional area of the sample. Young’s modulus was determined from the slope of the initial parts of the stress–strain curves. Hysteresis was estimated using the area under the cyclic stress–strain curves.

**Rheological measurements**: Rheological measurements were performed on a MCR302e rheometer (Anton Paar, Austria) equipped with 8 mm diameter stainless steel plate and a UV light system. Frequency sweep measurements were performed under an applied strain of 0.5%. The oscillatory shear mode was used to determine the storage modulus (G') and loss modulus (G"). The loss factor tan δ, was calculated from the ratio of G" to G'.

**Normal force measurements**: The UV-driven normal force (*F*_Ʇ_) was measured on the MCR302e rheometer (Anton Paar, Austria). As shown in Figure 4b, a small force was applied on the film, which was fixed on the quartz glass stage of the rheometer. The molecular orientation in the film is parallel to the stage and the force applied from top of the film is perpendicular to the molecular orientation. Then the change of the force before and during UV light was continuously monitored. It was observed that the force increased when UV was on and decreased when UV was turned off, indicating that the film thickness expanded because the gap distance was maintained constant throughout the tests. This expansion occurred in the direction perpendicular to the molecular orientation in the film. The adhesion force was measured using a probe tack test, conducted with either a manually designed apparatus or a rheometer equipped with a probe. The tests were performed at a constant speed of 1 mm/s or at several specified debonding speeds.

**UV–Vis absorption spectra**: The spectra were recorded using a JASCO V-670 spectrophotometer (JASCO). For the solution state, azo-C12 compounds were dissolved in CHCl_3_ and CHCl_3_ as the blank. For the polymer film, it was placed on a thin quartz glass, using the quartz glass as blank. The sample was irradiated with UV for different durations.

**Supplementary Figures**

Figure S1. Optical properties of DGI monomers. (a) Optical microscope images of DGI monomers (crystals). (b) Photograph and transmittance spectrum (inset) of melted DGI monomers sandwiched between two pieces of glass, exhibiting high optical transmittance from 300 nm to 600 nm.

Figure S2. Differential scanning calorimetry (DSC) thermographs of the azo-C12 monomers (three samples, synthesized at different times) during the first and second heating/cooling cycles at a constant rate of 5°C/min.

In the DSC analysis, the first heating of liquid crystal (LC) molecules typically shows a melting peak, which corresponds to the transition from a solid or lower-temperature phase to an isotropic liquid phase. During this first heating, the LC phase melts into an isotropic liquid, which is observed as the melting peak. In the second heating, after the material has fully melted, a phase transition peak appears as the LC molecules reorganize into a specific smectic LC phase with increasing temperature. This transition occurs due to the altered thermal history of the system, and during the second heating, the molecules have the opportunity to return to their LC phases from the isotropic liquid state.

Figure S3. Polarizing optical microscope (POM) observation of the smectic LC monomers. (a-b) POM images of the azo-C12 monomers, with an enlarged image shown, reveal a fan-shaped focal conic texture, which is characteristic of the smectic phase in liquid crystals.^[51]^

Figure S4. DSC thermographs of (a) DGI monomers and (b) DGI/azo-C12 mixtures.

Figure S5. Chemical structures and molecular lengths of the monomers. Space-filling models and chemical structures of (a-b) azo-C12 (trans), (c-d) azo-C12 (cis), and (e-f) DGI bilayer. The molecular lengths (L) were calculated using ChemDraw 3D.

Figure S6. Stress–strain curves of the free-standing films under elongation before and after UV irradiation at tensile speeds of (a)1 mm/min, (b) 5 mm/min, and (c) 10 mm/min.

Figure S7. Experimental setup for measuring the effect of light irradiation on the viscoelastic behaviour of the polymer films.

Figure S8. Normal force (*F*_Ʇ_) was monitored during UV on/off switching cycles (UV on for 1.2 s, off for 1.2 s, repeat more than 2000 times) on a freshly prepared sample. During testing, the film thickness expanded when UV was on and contracted when it was off, causing *F*_Ʇ_ to increase (UV on) and decrease (UV off) for thousands of times. The change in *F*_Ʇ_ (~0.015 N) during UV on/off remained almost constant throughout cyclic testing, indicating that this crosslinked polymer film exhibits rapid and reversible expansion/contraction with high stability. The initial increase is due to the rising fraction of cis-isomers, followed by a gradual decrease due to cis-to-trans thermal relaxation.

Figure S9. Effect of azobenzene substituents on the adhesion properties of the films. (a) The chemical structure and ^1^H NMR spectrum of the synthesized az-C8. (b) DSC thermographs of azo-C8. (c) The film synthesized with azo-C8 under the same condition exhibited significantly low adhesion force compared to the film synthesized with azo-C12.


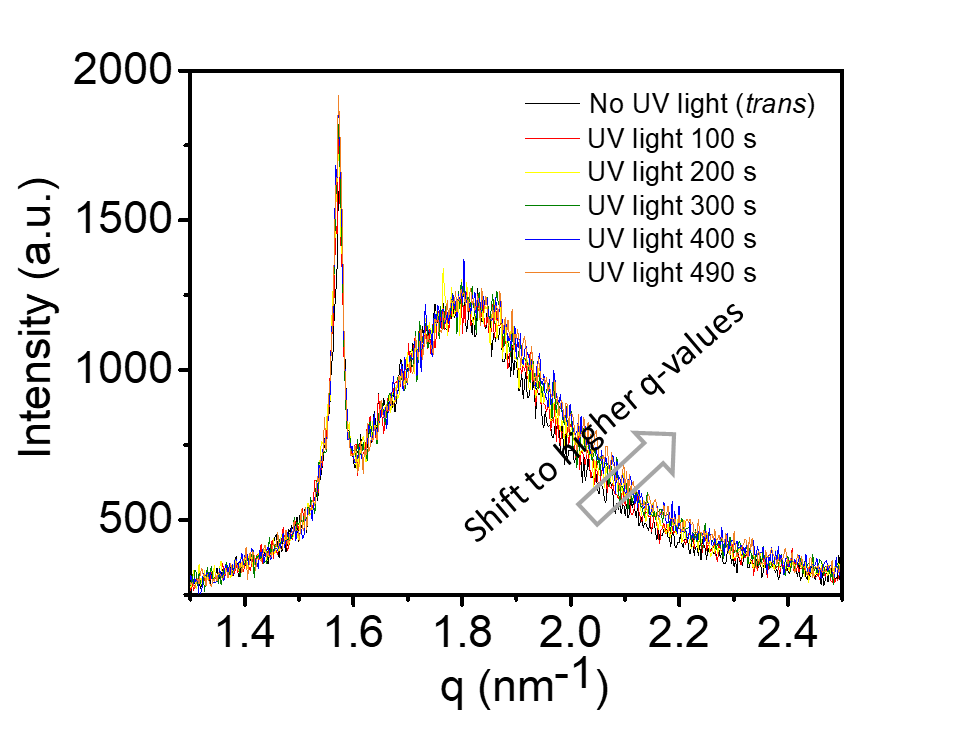


Figure S10. In-situ small angle X-ray scattering patterns of the films under UV light irradiation. The broad peak shift to higher q-values as the UV irradiation time increases.

Figure S11. The effect of irradiation light intensity on the light-triggered adhesion performance.

**
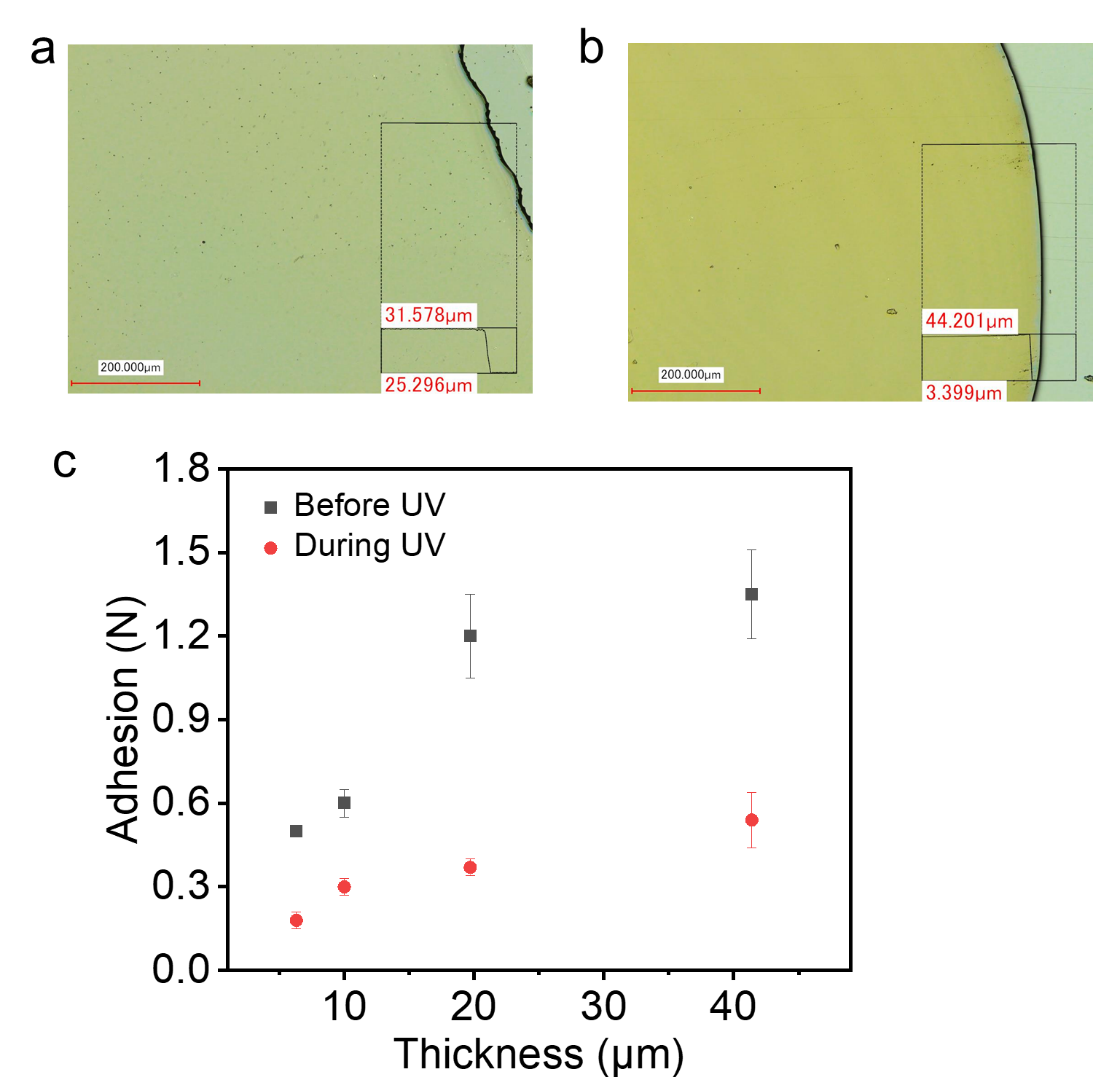
**

Figure S12. Effect of film thickness on light-triggered adhesion performance. (a) A thin film with thickness of 6 μm and (b) a thick film with thickness of 40 μm. (c) Light-responsive adhesion force of films with varying thicknesses.





Figure S13. Effect of crosslinking density on the light-responsive behaviour of the polymer films by controlling the crosslinker content (azo-C12). The films prepared with lower azo-C12 content (4.2 wt% and 11.5 wt%) were unable to form free standing polymer films. The adhesion fore decreased with increasing crosslinker content, and all the film exhibited decreased adhesion force upon UV light irradiation.

Figure S14. UV-vis absorption spectra of the thermal relaxation of the cis-azo state to the trans state in the film, measured at room temperature (25°C) in the dark. The results show that the cis-azo state of the film gradually returns to the trans state in the dark, requiring a long waiting time.

Figure S15. Temperatures of the film under UV light irradiation for different times, measured using an infrared thermometer camera. It indicates that a short time (e.g. < 10 s) UV light irradiation (365 nm, intensity: 98.6 mW/cm^2^) can hardly increase the temperature of the films.

Figure S16. Adhesion of the film to objects in different environments. (a) Adhesion behaviour of the film to various shapes and materials in air. (b) The film adhered with small items was immersed in water or ethanol.

**
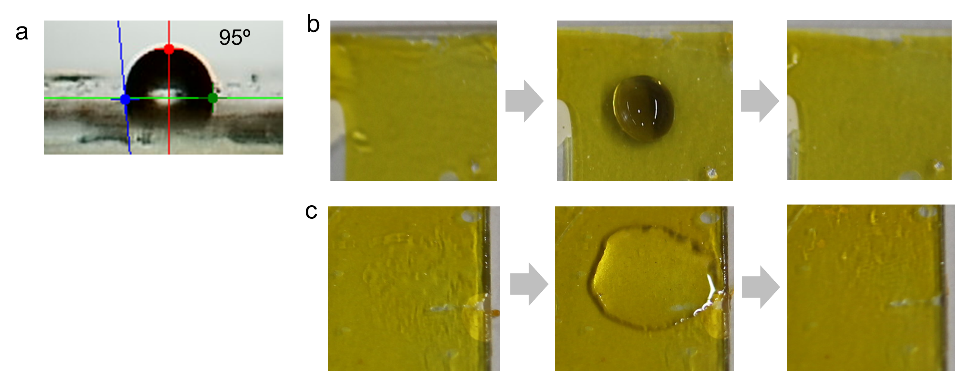
**

Figure S17. Anti-fouling performance of the film. (a) A high water contact angle (95º) indicates its resistance to aqueous contaminants. (b) A droplet of nanocarbon-dispersed water was placed on the film surface for 20 minutes. After water rinse, the contamination was effectively removed. (c) The film was exposed to silicone oil, a common hydrophobic contaminant. After rinsing with water, the oil residue was also removed. These results suggested that the film remain self-cleaning, preventing unwanted contamination from aqueous or oil-based substances.

Figure S18. Cis-contents of azo-C12 in solution and film state after UV irradiation. (a) UV-vis absorption spectra of azo-C12 (in CHCl_3_, 5*10^-5^M) before (0s, trans) and after UV irradiation (λ = 365 nm, 41.4 mW/cm^2^) for different times. (b) UV-vis absorption spectra of the film before UV (0s, trans) and after UV irradiation (λ = 365 nm, 41.4 mW/cm^2^) for different times. (c) Absorbance at 363 nm and estimated cis content in solution as a function of time. (d) Absorbance at 363 nm and estimated cis isomers in film as a function of time. The cis content was estimated by a method in literature.^[52]^ Cis content = (1-A/A_trans_)/(1-Ɛ_cis_/Ɛ_trans_), where A is the absorbance of azo-C12 in CHCl_3_ or in film, A_trans_ is the absorbance of trans in solution or film state, and Ɛ_cis_/Ɛ_trans_ ~0.05 for azobenzene.

**Supplementary Movies**

**Movie S1.** A movie showing polarizing optical microscope observation of the azo-C12 LC monomers upon cooling from 47 to 38.5 ºC.

**Movie S2.** A movie showing the adhesion and UV-driven detachment (debonding) of a ball.

**Movie S3.** A movie showing the adhesion and UV-driven selective release of balls one by one.

**Movie S4.** A movie showing the vis light cannot detach the ball efficiently.

**Movie S5.** A movie showing the film repeatedly lifted metallic alumina up and down in ethanol.
